# Genome-wide profiling of mouse RNA secondary structures reveals key features of the mammalian transcriptome

**DOI:** 10.1186/s13059-014-0491-2

**Published:** 2014-10-17

**Authors:** Danny Incarnato, Francesco Neri, Francesca Anselmi, Salvatore Oliviero

**Affiliations:** Human Genetics Foundation (HuGeF), via Nizza 52, Torino, 10126 Italy; Dipartimento di Biotecnologie Chimica e Farmacia, Università di Siena, via Fiorentina, Siena, 1-53100 Italy; Dipartimento di Scienze della Vita e Biologia dei Sistemi, Università di Torino, Via Accademia Albertina, Torino, 13-10123 Italy

## Abstract

**Background:**

The understanding of RNA structure is a key feature toward the comprehension of RNA functions and mechanisms of action. In particular, non-coding RNAs are thought to exert their functions by specific secondary structures, but an efficient annotation on a large scale of these structures is still missing.

**Results:**

By using a novel high-throughput method, named chemical inference of RNA structures, CIRS-seq, that uses dimethyl sulfate, and N-cyclohexyl- N'-(2-morpholinoethyl)carbodiimide metho-p-toluenesulfonate to modify RNA residues in single-stranded conformation within native deproteinized RNA secondary structures, we investigate the structural features of mouse embryonic stem cell transcripts. Our analysis reveals an unexpected higher structuring of the 5′ and 3′ untranslated regions compared to the coding regions, a reduced structuring at the Kozak sequence and stop codon, and a three-nucleotide periodicity across the coding region of messenger RNAs. We also observe that ncRNAs exhibit a higher degree of structuring with respect to protein coding transcripts. Moreover, we find that the Lin28a binding protein binds selectively to RNA motifs with a strong preference toward a single stranded conformation.

**Conclusions:**

This work defines for the first time the complete RNA structurome of mouse embryonic stem cells, revealing an extremely distinct RNA structural landscape. These results demonstrate that CIRS-seq constitutes an important tool for the identification of native deproteinized RNA structures.

**Electronic supplementary material:**

The online version of this article (doi:10.1186/s13059-014-0491-2) contains supplementary material, which is available to authorized users.

## Background

The development of high-throughput methods for the analysis of the epigenome and transcriptome have led to the discovery of thousands of previously unannotated transcripts [1,2], many of which lack the ability to encode proteins [3-6], as further proven by genome-wide ribosome profiling approaches [7]. While mechanisms of action have been elucidated for a small fraction of these non-coding RNAs (ncRNAs), for most the ways by which they contribute to gene regulation still remain unclear. One of the most intriguing modes of action proposed for long ncRNAs (lncRNAs) is their potential to act as modular scaffolds for the assembly of large multi-protein complexes [4,8], although the mechanistic aspects of these interactions are largely unknown. As learned from small nuclear ribonucleic particle (snRNP) complexes [9,10], most ncRNAs are thought to exert their functions by folding into locally stable secondary structures that may provide anchoring sites for interacting proteins. For example, it has been shown in *Drosophila melanogaster* that both MLE and MSL2 proteins of the MSL complex act by binding to conserved structural domains of the *roX1/2* ncRNAs, which then mediate targeting to the X chromosome to regulate dosage compensation in fruitfly [11,12]. Furthermore, in differentiating mouse embryonic stem cells (ESCs), MLL1 protein has been shown to be required for the transcriptional activation of *Hoxa6/7* genes, and its recruitment to chromatin is mediated by interaction with a stem-loop structure located in the 3′ region of the *Mistral* lncRNA [13].

The growing number of annotated transcripts has outpaced the efficient analysis of their structure; at present, structural information exists for only a very tiny minority of annotated RNAs. To address this need, over the past few years various enzymatic- and chemical-based approaches have been proposed for the discovery of secondary structures for thousands of RNAs at a time [14-18]; however, all these methods are based on the assumption that *in vitro* folding may be representative of native RNA structures *in vivo*. While for certain small RNAs the *in vitro* folding landscape recapitulates well the *in vivo* one [19-21], long RNAs often exhibit rugged folding landscapes that lead *in vitro* to the prevalence of kinetically trapped intermediates and misfolded structures [22-24]. For example, *in vitro* folding of the RNAse P ribozyme is a slow process that takes several minutes and requires escape from a kinetic trap [23,25]. Comparative analysis of *in vivo* and *in vitro* probing data on human telomerase RNA revealed that while the 3′-terminal small nucleolar RNA (snoRNA)-like domain folds into comparable structures in the two conditions, the 5′ template domain exhibits very different foldings [26].

Two main scenarios can explain the differences observed in RNA folding *in vitro* and *in vivo*. The first is based on the assumption that, in the cell, most nascent transcripts are likely to fold during transcription [20,27,28]. In this perspective, the elongation rate of RNA polymerase, as well as the directionality of transcription, may influence the order and the speed of the folding events, thus preventing the formation of non-native, kinetically trapped intermediates [29]. The second, which does not exclude the first, is that many specific as well as non-specific RNA binding proteins (RBPs) may act as RNA chaperones, thus directing and stabilizing RNA folding [30-33]. To overcome the issues introduced by the study of RNA folding *in vitro*, two recent reports analyzed the structures of *Saccharomyces cerevisiae* and *Arabidopsis thaliana* RNAs by treating the cells with dimethyl sulfate (DMS) [34,35].

We present here a new method, named chemical inference of RNA structures followed by massive parallel sequencing (CIRS-seq), that allows genome-wide investigation of native deproteinized RNA secondary structures by exploiting the capacity of DMS and N-cyclohexyl-N’-(2-morpholinoethyl)carbodiimide metho-p-toluenesulfonate (CMCT) to specifically react with RNA unpaired bases. Our approach, applied to mouse ESCs, allowed us to obtain single-base resolution structural information for thousands of transcripts in their native deproteinized conformation, revealing the structural complexity of the mammalian transcriptome.

## Results

### CIRS-seq enables accurate transcriptome-wide inference of single-stranded RNA residues

**Figure 1:**
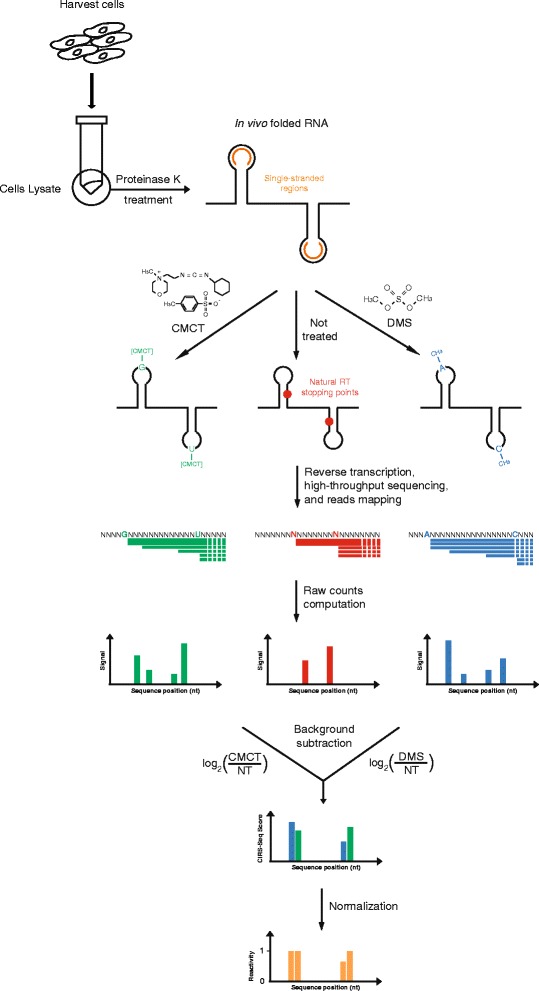
**Overview of the CIRS-seq method.** Cells are harvested and lysed in isotonic buffer, then treated with Proteinase K to unmask protein-bound regions of RNAs. The whole cell population of RNAs in their native deproteinized conformation is probed with either DMS or CMCT to modify unpaired bases. A non-treated control is also produced to allow further mapping of natural RT stops. After modification, the RNAs from the three populations are reverse transcribed, and cDNA is adapter ligated for high-throughput sequencing. Mapping reads to the transcriptome provide information regarding how many RT stops occurred at each position of the analyzed transcripts. The non-treated (NT) signal at each position is then subtracted from the DMS and CMCT signals to obtain the raw reactivity profile at base resolution. After scaling each data point above the 90th percentile to the 90th percentile, reactivity at each position is divided by the 90th percentile (90% Winsorising) to obtain the normalized reactivity.

The CIRS-seq method (Figure 1) is based on the use of DMS, which mainly methylates N1 of adenosine and N3 of cytosine [36,37], and CMCT, which primarily forms adducts with N1 and N3 of pseudouridine, N3 of uridine, and, to a lesser extent, N1 of guanosine and inosine [38-40] but only when these residues are in single-stranded conformation. Treatment of RNA with the two reagents enables the detection of unpaired nucleotide positions due to the modification-induced reverse transcription (RT) stop one nucleotide downstream of the modified residue. To carry out CIRS-seq, we first optimized treatments to achieve similar degrees of modification with the two reagents at different concentrations, as measured by reduction of the full-length reverse transcription product for a test RNA following reaction with either DMS or CMCT (Figure S1 in Additional file 1).

To perform transcriptome-wide probing of RNAs in their native deproteinized conformation, we lysed mouse ESCs in an isotonic buffer, and treated the lysate with Proteinase K to unmask regions of RNAs bound by proteins, without affecting the RNA structure (supplementary Materials and methods in Additional file 1). ESC lysates were then treated with DMS or CMCT, and total RNA was extracted following reaction quenching. Extracted RNA was subjected to random-primed RT. A non-treated control was also produced to determine naturally occurring RT stops. The generated cDNAs were adapter ligated and subjected to high-throughput sequencing using the Illumina platform, resulting in about 90 × 10^6^ deep-sequencing reads for each treatment, across two biological replicates.

**Figure 2:**
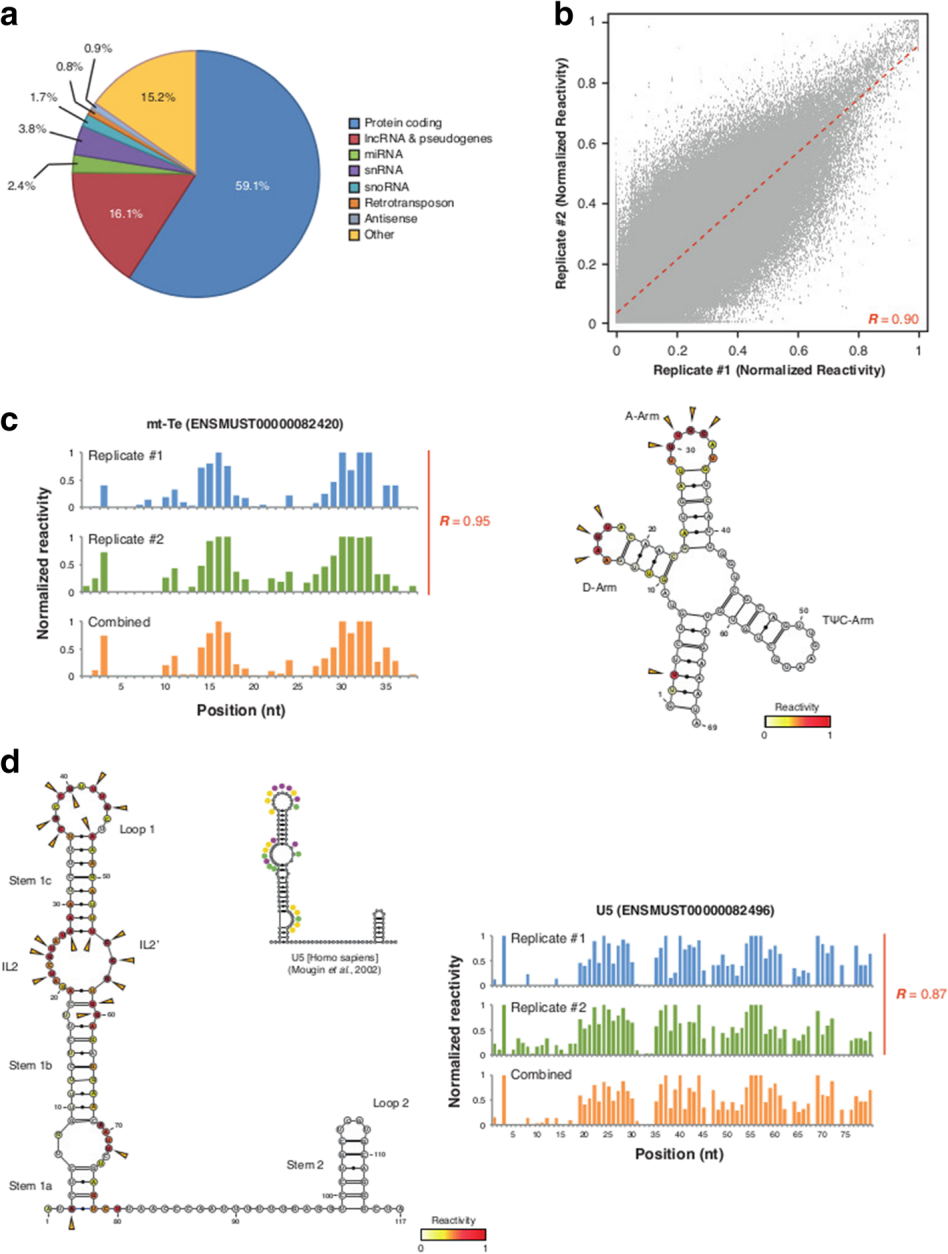
**Validation of CIRS-seq data. (a)** Distribution of transcripts with at least one RT stop on average per base. **(b)** Scatter plot of normalized reactivities in the two biological replicates of CIRS-seq. Reactivities are averaged in 10-nucleotide windows, with an offset of 5 nucleotides (Pearson’s correlation coefficient = 0.90. **(c)** Normalized reactivity profiles for the glutamic acid tRNA and overlay of reactivity data on the phylogenetically derived secondary structure. Yellow arrows indicate highly reactive positions (reactivity >0.7). Bases are color coded according to their reactivity. **(c)** Normalized reactivity profiles for the U5 snRNA and overlay of reactivity data on phylogenetically derived secondary structure. The structure of the U5 human homolog is also shown, with superimposed DMS/CMCT-reactive positions from [55]. The colors correspond to different degrees of chemical modification (purple, strong; yellow, medium; green, weak). Yellow arrows indicate highly reactive positions (reactivity >0.7). Bases are color coded according to their reactivity.

Since proper analysis of RNA folding requires correct annotation of transcript sequences, reads were mapped to a recently published variant of the mm9 assembly that integrates single-nucleotide variants from the E14 ESC line [41], and we obtained a similar distribution of read mappings across all samples (Figure S2A in Additional file 1). Estimation of transcript abundances using CIRS-seq data correlated well across treatments, and with canonical RNA-seq data (*R* ≥0.9, Spearman correlation; Figure S2B in Additional file 1), showing that the CIRS-seq method enables unbiased probing of RNAs. At the current coverage, we obtained structural information for approximately 30,000 transcripts, belonging to approximately 13,000 genes (Figure 2a; Figure S2C in Additional file 1).

As a quantitative measure of the probability of observing a RT stop specifically induced by our treatment, we calculated raw reactivity scores as the base 2 logarithm ratio of the normalized read counts for the DMS/CMCT treatment at a given position of a transcript, and the normalized read counts at the same position in the non-treated control. The final normalization (Supplementary methods in Additional file 1) yielded reactivity values ranging from 0 to 1, and positions with reactivities >0 and <0.3, 0.3 to 0.7, or >0.7 were designated as weakly, moderately, or highly reactive, respectively [42]. Correlation analysis of reactivity values across the top 75th percentile of covered transcripts revealed the high reproducibility of CIRS-seq (*R* = 0.90, Pearson correlation; Figure 2b); therefore, we combined the two replicates for further analysis.

Collectively, we obtained structural data for 1,190,948 and 1,080,859 nucleotides in the DMS (weak, 13.6%; moderate, 49.9%; high, 36.5%) and CMCT (weak, 16.6%; moderate, 54.7%; high, 28.7%) treatments, respectively (Figure S2D in Additional file 1). To validate CIRS-seq, we overlaid reactivity data on the well characterized structures of tRNAs [43,44] (Figure 2c; Figure S3A,B in Additional file 1), and observed that all the highly reactive residues were almost completely confined to the tRNAs' D and anticodon arm loops, suggesting a high overall accuracy for our method.

Despite the respective strong preference of DMS and CMCT for A/C and G/U residues, we also observed non-canonical reactivities in both treatments. Our data are in agreement with previous reports showing DMS reactivity with G/U residues [36,45-47] and CMCT reactivity with cytosines [48-50]. We observed a significant increase in the accuracy of the *de novo* prediction of structures when considering also these non-canonical reactivities as both the canonical and non-canonical reactivities lay within single-stranded regions (Figure S3C in Additional file 1). Moreover, overlaying reactivity data on the known structures of U5 and U1 small nuclear RNAs (snRNAs) and U3 snoRNA (Figure 2d; Figure S4A,B in Additional file 1) showed that the Proteinase K treatment enabled high-resolution determination of secondary structures at the level of protein-masked regions of RNAs without losing the proper folding. In fact, internal loop IL2/IL2′ of U5, box B/C of U3 and loop II of U1 are bound *in vivo* by, respectively, a 116 kDa protein (Snu114p yeast homolog) [51], a 15.5 K protein [52,53], and the U1A protein [10]; these regions showed very high reactivity to DMS/CMCT treatments and were almost completely resolved by CIRS-seq. Overall, for the set of analyzed structures (Table 1), 80.6% of the highly reactive residues were located within single-stranded regions. Of the 19.4% of the highly reactive residues located within regions of the known structures annotated as double-stranded, 84.2% were positioned at the end of helices or adjacent to bulges/loops. These regions were previously shown to be subjected to structural flexibility, so chemical reagents can easily modify these terminal residues [34,54]. When accounting for these additional accessible positions, the overall true positive rate of our method rose to 96.3%.

**Table 1:** **CIRS-seq efficiency on validated secondary structures**

		**Including helix termini**		**Excluding helix termini**	
**ENSEMBL ID**	**Symbol**	**TP (%)**	**FP (%)**	**TP (%)**	**FP (%)**
ENSMUST00000082420	mt-Te	87.5	12.5	87.5	12.5
ENSMUST00000082389	mt-Ti	75.0	25.0	100.0	0.0
ENSMUST00000082399	mt-Tn	90.0	10.0	90.0	10.0
ENSMUST00000083033	U1	83.4	16.6	94.7	5.3
ENSMUST00000082496	U5	73.7	26.3	100.0	0.0
ENSMUST00000082466	U3	75.0	25.0	96.9	1.1
Total		80.6	19.4	96.3	3.7

Percentages of true positive (TP) and false positive (FP) highly reactive positions for known secondary structures.

Collectively, this analysis proves the high accuracy of CIRS-seq, and provides a nucleotide-resolution panorama of the mouse ESC RNA structurome.

### CIRS-seq data allow accurate secondary structure prediction

**Figure 3:**
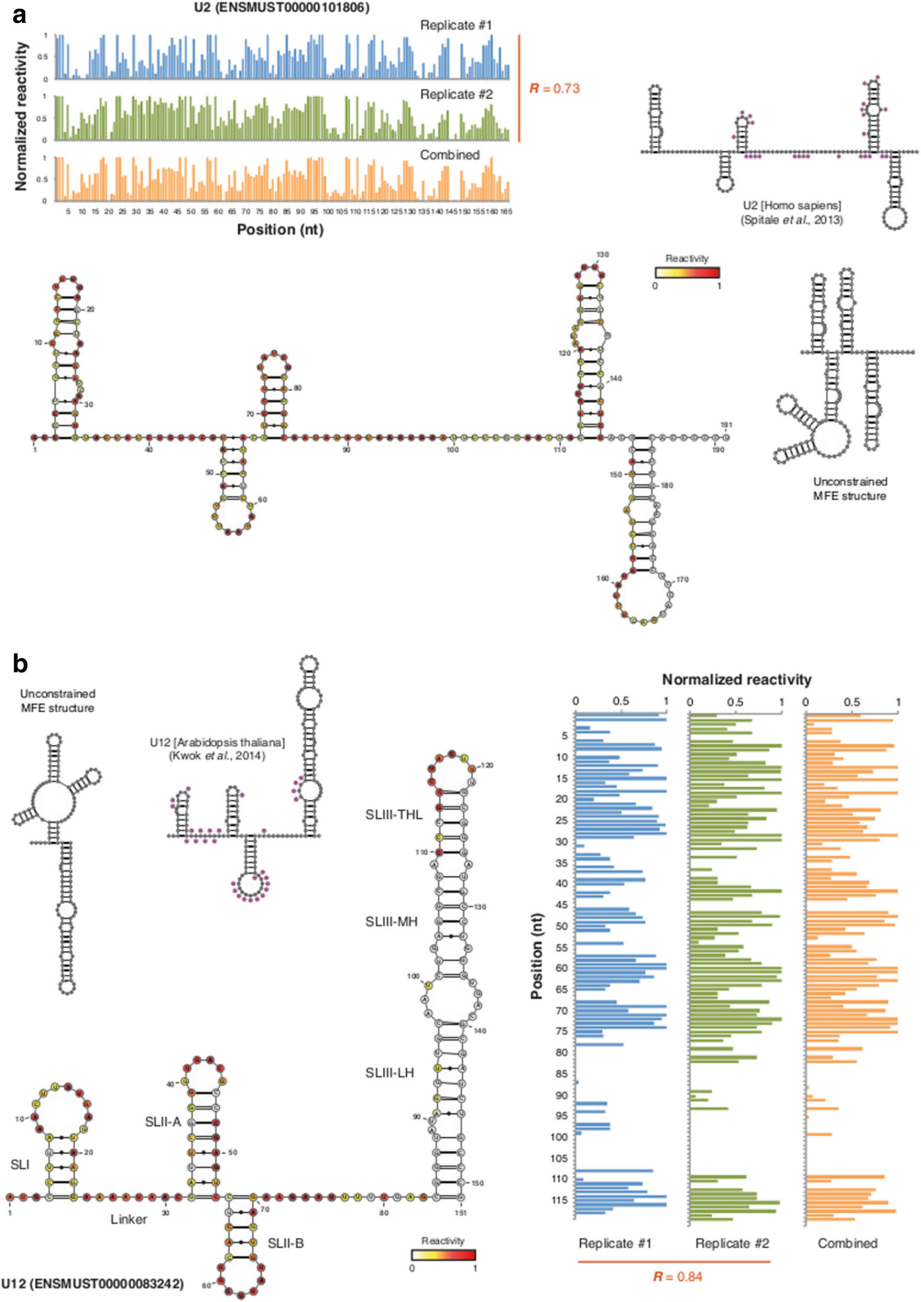
**CIRS-seq data allow correct inference of native deproteinized RNA secondary structures. (a)** Normalized reactivity profiles for the U2 snRNA and overlay of reactivity data on the secondary structure inferred from chemical constraints. Bases are color coded according to their reactivity. The structure of the human ortholog with superimposed SHAPE-reactive positions from [57], and the unconstrained MFE structure are also shown. **(b)** Normalized reactivity profiles for the low-abundance U12 snRNA and overlay of reactivity data on the secondary structure inferred from chemical constraints. Bases are color coded according to their reactivity. The structure of the U12 *A. thaliana* ortholog with superimposed DMS/SHAPE-reactive positions from [58], and the unconstrained MFE structure are also shown.

Next, we verified the ability of CIRS-seq to infer *de novo* secondary structures. Constraints derived from chemical probing data may significantly improve the accuracy of RNA secondary structure prediction tools [42,56]. We chose the U2 and low-abundance U12 snRNAs, and the valine and threonine tRNAs, whose structures were previously experimentally defined [57,58], or can be easily derived from phylogenetic analysis. We used the RNAStructure tool [59] to devise secondary structures by imposing constraints for unpaired positions. This tool can accept chemical probing data in the form of SHAPE data files, allowing more comprehensive modeling of the structure according to the CIRS-seq-derived data compared with hard constraints-based methods. For both the unconstrained minimum free energy (MFE) and the CIRS-seq constrained secondary structures, we calculated the positive predictive value (PPV) as the fraction of base-pairs present in the predicted structure that are also present in the validated structure, and the sensitivity as the fraction of base-pairs present in the validated structure that are also in the predicted structure (Table 2). Notably, CIRS-seq-derived structures for all the four transcripts analyzed showed higher similarity to the known structures (Figure 3a,b; Figure S5A,B in Additional file 1); on average, the CIRS-seq-guided folding outperformed the MFE unconstrained predictions in terms of both PPV and sensitivity (PPV 0.53 and sensitivity 0.57 for unconstrained MFE structures; PPV 0.95 and sensitivity 0.95 for CIRS-seq constrained structures). This analysis demonstrates that the use of CIRS-seq data improves the accuracy of RNA secondary structure prediction tools, and that low-abundance transcripts can be successfully probed by CIRS-seq.

**Table 2:** **Statistics for CIRS-seq** ***de novo*** **inferred secondary structures**

		**Unconstrained (MFE)**		**CIRS-seq constrained**	
**ENSEMBL ID**	**Symbol**	**PPV**	**Sensitivity**	**PPV**	**Sensitivity**
ENSMUST00000101806	U2	0.68	0.89	1.00	1.00
ENSMUST00000083242	U12	0.80	0.84	1.00	0.95
ENSMUST00000082389	mt-Tv	0.22	0.20	1.00	1.00
ENSMUST00000083422	mt-Tt	0.41	0.35	0.81	0.85
Average		0.53	0.57	0.95	0.95

Positive predictive value (PPV) and sensitivity measures calculated for both the unconstrained minimum free energy (MFE) and CIRS-seq constrained structures.

### CIRS-seq reveals structural features of mammalian mRNAs and ncRNAs

Thanks to the high resolution enabled by CIRS-seq, we then investigated the structural features of mouse mRNAs, and looked for structural differences across transcript regions. We selected approximately 9,500 mRNAs, in which the DMS/CMCT treatment induced, on average, at least one RT stop per nucleotide (Supplementary methods in Additional file 1).

**Figure 4:**
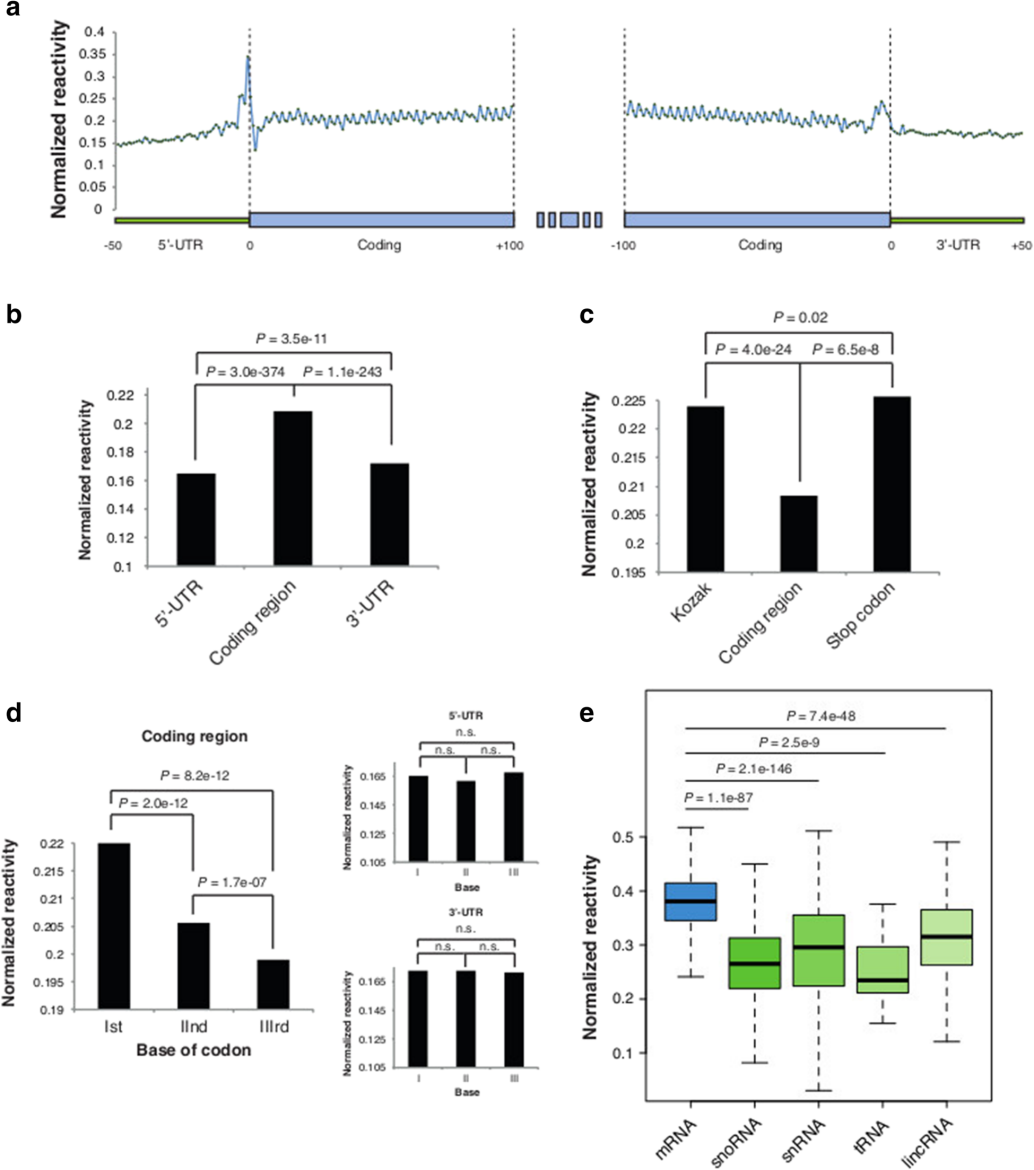
**Transcriptome-wide analysis of mRNAs reveals structural features of protein-coding and non-coding transcripts. (a)** Meta-gene analysis across the last 50 nucleotides of the 5′ UTR, the first and last 100 nucleotides of the coding region, and the first 50 nucleotides of the 3′ UTR of approximately 9,500 mRNAs. **(b)** Average reactivity of the 5′ UTR, coding region, and 3′ UTR. **(c)** Average reactivity on the Kozak sequence (−6/+1 nucleotides around AUG), coding region, and stop codon (+3 nucleotides upstream). **(d)** Average reactivity for the first, second, and third base of each coding sequence codon, and for the first, second, and third base of the 5′ UTR and 3′ UTR, respectively, in the first and last 99 nucleotides of the coding region, last 48 nucleotides of the 5′ UTR, and first 48 nucleotides of the 3′ UTR. **(e)** Box-plot of base-normalized average CIRS-seq reactivities for protein-coding and non-coding RNAs, calculated on all transcript positions with sequencing depth >50 × .

Meta-analysis of average reactivity across UTRs and coding regions revealed a strong reduction of reactivity scores in the 50 nucleotides of the 5′ UTR immediately preceding the Kozak sequence (average 0.165) compared with the first and last 100 nucleotides of the coding region (average 0.208, *P*-value 3.0e-374, Wilcoxon rank sum test; Figure 4a,b). Moreover, a significant reduction of reactivity was also observed in the first 50 nucleotides of the 3′ UTR immediately downstream of the stop codon (average 0.172, *P*-value 1.1e-243, Wilcoxon rank sum test). These results differ from what has been recently observed in *A. thaliana*, where the coding region is more structured than the UTRs [34]. We also identified a significant increase of reactivity score at the level of the Kozak sequence (average 0.229) with a maximum of reactivity on the base immediately preceding the AUG (average 0.345), and on the stop codon beginning three nucleotides upstream (average 0.226), compared with the coding region (*P*-values 4.0e-24 and 6.5e-8, respectively, Wilcoxon rank sum test; Figure 4c), revealing a markedly reduced probability of base-pairing in these regions. The reduced base-pairing on the Kozak sequence and around the stop codon suggests that a more accessible context in these regions of protein-coding transcripts may facilitate both the entry and the detachment of ribosomes.

We next analyzed the first and last 99 nucleotides of mRNA coding regions to determine if the previously reported periodic signal of three nucleotides [14,34] was conserved also in mouse. To this end, we observed that mouse protein-coding transcripts, similar to *A. thaliana* and *S. cerevisiae* mRNAs, exhibit a strong three-nucleotide periodicity across the coding region that was not observed within the UTRs (Figure 4d). The second and third nucleotides of each codon were highly structured and exhibited lower average reactivities (average 0.205 and 0.199, respectively), with the third nucleotide being the less reactive (*P*-value 1.7e-07, Wilcoxon rank sum test), while the first nucleotide was the less structured and significantly more reactive to DMS/CMCT treatment than the second and third (average 0.220, *P*-values 2.0e-12 and 8.2e-12, respectively, Wilcoxon rank sum test). Taken together these results suggest a deep involvement of RNA secondary structures in driving and regulating translation efficiency.

Analysis of the RNA structure is particularly relevant for ncRNAs as they are thought to exert their function by interacting with other molecules via their secondary structure. We then sought to determine whether an overall structural difference exists between protein coding RNAs and different classes of ncRNA transcripts. To avoid biases due to differential coverage, only transcript positions with sequencing depth greater than 50× were considered (Supplementary methods in Additional file 1). Analysis of normalized reactivity showed a significantly lower average reactivity of snoRNAs (average 0.282, *P*-value 1.1e-87, Wilcoxon rank sum test), snRNAs (average 0.295, *P*-value 2.1e-146, Wilcoxon rank sum test), tRNAs (average 0.251, *P*-value 2.5e-9, Wilcoxon rank sum test), and long intergenic non-coding RNA (lincRNAs; average 0.309, *P*-value 7.4e-48, Wilcoxon rank sum test) compared with mRNAs (average 0.366) (Figure 4e). Collectively, these data reveal a higher structuring of ncRNA transcripts compared with mRNAs.

### CIRS-seq identifies structural requirements of RNA binding proteins

**Figure 5:**
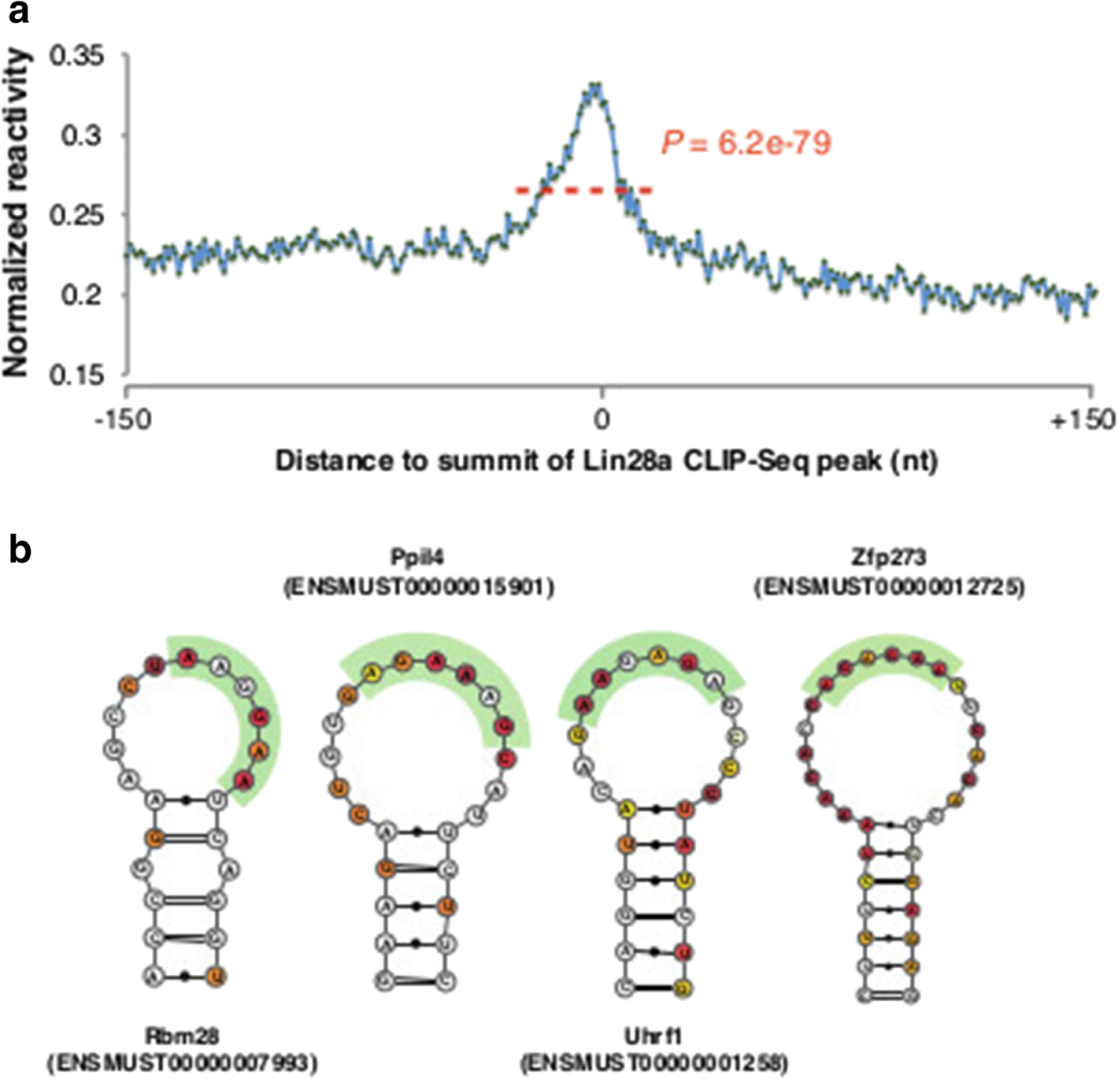
**CIRS-seq reveals structural preferences of RNA binding proteins. (a)** Average reactivity across 300 nucleotides surrounding summits of Lin28a peaks. **(b)** Representation of sample secondary structures for Lin28a binding sites. Bases are color coded according to their reactivity. The purine-rich motifs are highlighted in green.

RNA-protein interactions are strongly influenced by secondary structures. Determining the structural requirements for RBPs to bind to their cognate targets is required to understand their roles and mechanisms of action. To this end, we analyzed from a structural perspective the binding sites of the highly conserved RBP Lin28a. Lin28a is highly expressed in ESCs, and is one of the factors required for the reprogramming of human fibroblasts to induced pluripotent stem cells [60]. To investigate the structural requirements of Lin28a binding, we analyzed a previously published CLIP-seq dataset of Lin28a in ESCs [61]. We identified peaks of Lin28a enrichment across the mouse transcriptome, and calculated average reactivity on a window of 300 nucleotides surrounding summits of the peaks (Figure 5a). While more distal regions around the Lin28a peaks showed a level of reactivity comparable to that of the coding sequence (average 0.21), in agreement with a preferential positioning of Lin28a binding sites within this region, we observed a significant and progressive increase in the accessibility proceeding toward the peak summits (maximum 0.34, ±25 nucleotides average 0.27, *P*-value 6.2e-79, Wilcoxon rank sum test). Concordant with this observation, analysis of putative Lin28a binding sites revealed that the target A/G-rich motifs tends to assume a single-stranded conformation within the loop regions of hairpin-like structures (Figure 5b). This result is in agreement with previous *in silico* predictions based on the analysis of Watson-Crick pair co-occurrence around the Lin28a consensus [61].

## Discussion

In this work we have defined, for the first time, the complete RNA structurome of mouse ESCs. Our analysis revealed the structural features of mRNAs and ncRNAs, and identified the structural requirements for Lin28a RNA binding protein.

The introduction of the CIRS-seq method, which does not rely on a denaturation and re-folding approach, allowed us to massively probe RNAs in their natural context. By applying CIRS-seq to mouse ESC RNAs, we were able to probe protein-coding RNAs as well as ncRNAs in their native deproteinized conformation. Analysis of previously validated secondary structures showed that CIRS-seq is extremely precise, and RNA secondary structures inferred using CIRS-seq data to constrain folding algorithms exhibit higher accuracy than MFE structures predicted in the absence of chemical probing data. Moreover, the use of two compounds that modify distinct bases, together with the introduction of a deproteinization step, which enabled us to investigate protein-masked regions of transcripts without losing their correct folding, increased the resolution of our method.

The analysis of CIRS-seq data revealed a strong structural partitioning of protein-coding transcripts, revealing a higher degree of structuring of UTRs compared with coding regions. This was unexpected since it has been recently reported that in *A. thaliana* UTRs have a higher propensity to single-strandedness than coding regions [34]*.* This difference may represent evolutionary structural diversity between metazoans and plant RNAs, as suggested by previous *in vitro* [17] and *in silico* [62] analyses, or it could be explained by reduced accessibility of transcript coding regions to DMS treatment, due to the ribosome occupancy, in the absence of a deproteinization step. However, the agreement of our data with a recent nuclease-based analysis conducted in human lymphoblastoid cells [63] suggests that this structuring is conserved in mammalian mRNAs and may have a functional role.

The slightly higher reactivity observed for 3′ UTRs compared with 5′ UTRs in mouse mRNAs may be representative of the preference of microRNA recognition elements, which are highly enriched in 3′ UTRs [64], to reside within more accessible contexts [65,66]. It must be also noticed that structural regulatory elements in the 3′ UTR are often short and dispersed in the UTR, which in many cases may be very long, thus leading to a lower overall structuring of this region compared with the 5′ UTR [67].

Our analysis of Lin28a protein recognition elements demonstrated genome-wide that binding sites for this protein tend to preferentially assume a single-stranded conformation. We moreover observed that Lin28a motifs tend to reside within loop regions of hairpin-like structures.

Furthermore, the analysis of ncRNAs revealed a higher overall degree of structuring compared with protein-coding transcripts, and showed that lincRNAs exhibit structural features intermediate to those of mRNAs and structural ncRNAs. This is in agreement with the report that ncRNAs have higher melting temperatures than mRNAs, denoting higher structural stability [18].

Collectively, our data demonstrate that CIRS-seq can be used to obtain genome-wide information on native deproteinized RNA structures. Moreover, CIRS-seq methodology represents an important tool for the study of the structural binding specificities of RBPs.

## Conclusions

We define for the first time the complete RNA structurome of mouse ESCs, by developing a high-throughput method for the analysis of RNA secondary structures in their native deproteinized conformation. This method achieved extremely high accuracy on validated secondary structures, and allowed the *de novo* prediction of RNA structures. Analysis of structural data for protein-coding RNAs revealed their strong structural partitioning between 5′ UTRs, coding sequences, and 3′ UTRs. Comparison with non-coding RNAs showed that ncRNAs are more structured than mRNAs, and that lincRNAs present an average structuring midway between protein coding and structural non-coding transcripts. We also reveal the structural requirements for binding of the RBP Lin28a, and demonstrate that our method can provide insight into the structural preferences of RBPs.

## Materials and methods

### Cell culture

Mouse E14 ESCs were grown on 0.1% gelatin-coated plates and maintained in DMEM (4.5 g/L D-glucose) supplemented with 15% heat-inactivated fetal bovine serum, 0.1 mM NEAA, 1 mM sodium pyruvate, 0.1 mM 2-mercaptoethanol, 25 U/ml penicillin, 25 μg/ml streptomycin and 1,500 U/ml LIF, as previously described [68].

### Quantitative RT-PCR

Real-time quantitative PCR was performed using the SuperScript III Platinum One-Step Quantitative RT- PCR System (Invitrogen Carlsbad, CA, USA) as previously described [69]. The primers for the Rpph1 test transcript are provided in Table S1 in Additional file 1.

### RNA-seq library preparation

For RNA-seq library preparation, approximately 1 μg of TRIzol (Invitrogen) isolated total RNA from ESCs was subjected to ribosomal RNA depletion using the Ribo-Zero Gold Kit (Epicentre Madison, Winsconsin, USA). rRNA-depleted RNA was used as the input for the RNA-seq library preparation using the TruSeq RNA Sample Prep Kit (Illumina) following the manufacturer’s instructions.

### CIRS-seq

Cell lysis and chemical probing, library preparation, and sequencing are detailed in the supplementary Materials and methods in Additional file 1.

### RNA quality assessment

RNA sample quality was assessed with the Agilent Bioanalyzer 2100. All of the samples had an RNA integrity number ranging from 9.9 to 10.

### Data analysis

CIRS-seq data analysis, normalization and background subtraction, and transcript analysis are detailed in the supplementary Materials and methods in Additional file 1.

### Data access

CIRS-seq and RNA-seq data have been deposited in the Gene Expression Omnibus (GEO) under accession number GSE54106. Additional datasets and the source code for the analysis tool are available at [70].

## Additional file

Additional file 1: **PDF file containing supplementary Materials and methods, Figures S1 to S5), Table S1, and supplementary references.**

## Abbreviations

CIRS: chemical inference of RNA structures
CMCT: N-cyclohexyl-N’-(2-morpholinoethyl)carbodiimide metho-p-toluenesulfonate
DMS: dimethyl sulfate
ESC: embryonic stem cell
lincRNA: long intergenic non-coding RNA
lncRNA: long non-coding RNA
MFE: minimum free energy
ncRNA: non-coding RNA
PPV: positive predictive value
RBP: RNA binding protein
RT: reverse transcription
snRNA: small nuclear RNA
snoRNA: small nucleolar RNA
UTR: untranslated region
