# Supplementary material for: Genome-wide profiling of mouse RNA secondary structures reveals key features of the mammalian transcriptome
Source: Genome Biol. 2014 Oct 17;15(10):491. doi: 10.1186/s13059-014-0491-2 (PMC4220049; doi:10.1186/s13059-014-0491-2)
Supplement: Supplementary file 1 — PDF file containing supplementary Materials and methods, Figures S1 to S5), Table S1, and supplementary references. [file 13059_2014_491_MOESM1_ESM.pdf]

Supplementary Information

Genome-wide profiling of mouse RNA secondary structures reveals key features of the mammalian transcriptome

Danny Incarnato, Francesco Neri, Francesca Anselmi, Salvatore Oliviero

SI Materials and Methods

Cells lysis and RNA probing

Approximately 10^7 cells were harvested and lysed in 1 ml of ice-cold Structure Buffer D (10 mM Hepes-KOH pH 7.9, 100 mM KCl, 10 mM $MgCl_2$, and 0.5% NP-40) for dimethyl sulfate (DMS) probing, or Structure Buffer C (50 mM potassium borate pH 8.0, 100 mM KCl, 10 mM $MgCl_2$, and 0.5% NP-40) for N-cyclohexyl-
N'-(2-morpholinoethyl)carbodiimide metho-p-toluenesulfonate (CMCT) probing, supplemented with 100 U/ml RNAse Inhibitor (Ambion), 100 U/ml RNAse OUT (Invitrogen), and 100 U/ml Superase IN (Invitrogen). After lysis, the extract was treated with 100 µg/ml of Proteinase K (Sigma) for 15 minutes at 30°C, and the reaction was stopped by adding 20 µl of Protease Inhibitor Cocktail (Sigma). The sample was then divided into 5 x 200 µl aliquots. For DMS treatment, DMS (Sigma) was added to final concentrations of 0, 50, 100, 150, and 200 mM to each aliquot, and the samples were incubated at 30°C for 2 minutes with moderate shaking. The reaction was quenched by placing the samples on ice and adding 0.7 M final concentration of ice-cold 2-mercaptoethanol and 1 ml ice-cold TRIzol (Invitrogen). After chloroform addition and centrifugation, one volume of 100% ethanol was added to the upper aqueous phase, and the sample was purified using RNEasy Mini Spin Columns (Qiagen) to allow complete removal of 2-mercaptoethanol and DMS. For CMCT treatment, CMCT (Sigma) was added to final concentrations of 0, 10, 20, 25, and 50 mM to each aliquot, and the samples were incubated at

30°C for 20 minutes with moderate shaking. The reaction was stopped by the addition of 1 ml ice-cold TRIzol.

CIRS-seq library preparation

For CIRS-seq library preparation, we first prepared pools of each treatment. The DMS sample was obtained by pooling the 50-100-150-200 mM-treated conditions, while the CMCT sample was obtained by pooling the 10-20-25-50 mM-treated conditions (~1.25 µg each). The samples obtained by treating with 0 mM DMS and 0 mM CMCT were pooled in equal amounts (~2.5 µg each) and constituted the Non-treated (NT) control. Ribosomal RNAs were depleted using the Ribo-Zero Gold Kit (Epicentre). One-third of each sample (corresponding to approximately 100 ng of Ribo- RNA) was subjected to reverse transcription with random hexamers using SuperScript II Reverse Transcriptase (Invitrogen). The reverse transcription reaction was conducted in 1 hour at 42°C, followed by 10 minutes at 70°C to inactivate the reverse transcriptase. The template RNA was then degraded by adding 10 U of Ribonuclease H (Ambion) and incubating for 20 minutes at 37°C. After ethanol precipitation of the cDNA, an adapter modified with a 5'-P group and a 3'-C3 spacer, corresponding to the reverse complement of the standard Illumina TruSeq Small RNA 5' adapter (RC5), was ligated to cDNA 3'-OH termini using 200 U of CircLigase II for 4 hours at 68°C. This approach allowed us to keep the strand-specificity of the library so that each read started 1 nt downstream of the RT stopping point. Then, to enable the ligation of a 5' adapter, the cDNA was treated with T4 Polynucleotide Kinase (NEB) in T4 DNA Ligase

Buffer (NEB) for 1 hour at 37°C. After ethanol precipitation, the cDNA was loaded on a TBE-Urea 5% PAGE gel. A gel slice corresponding to 70-200 nt was cut, and the cDNA was recovered by passive diffusion into diffusion buffer (500 mM ammonium acetate, 1 mM EDTA, 10 mM magnesium acetate, 0.1% SDS) for 16 hours at 37°C, followed by ethanol precipitation. The cDNA 5' termini were then ligated to a second adapter, corresponding to the reverse complement of the standard Illumina TruSeq Small RNA 3' adapter (RC3) using 200 U of CircLigase II for 4 hours at 68°C. The adapter-ligated cDNA was then subjected to 15-18 cycles of PCR using standard Illumina TruSeq primers. To remove the adapter dimers, the library was loaded on a 3% (w/v) TBE-agarose gel, and the slice corresponding to 150-300 nt was cut and purified using the NucleoSpin Gel and PCR Clean-up Kit (Macherey-Nagel).

Transcriptome assembly and reads mapping

For reads mapping, we used a recently published mm9 reference genome assembly variant that incorporates E14 ESC SNVs [1]. Prior to mapping, the reads quality was estimated using the FastQC tool v0.10.1 (http://www.bioinformatics.babraham.ac.uk/projects/fastqc/). The nucleotide positions with a quality score below 20 (Phred33 scale) were trimmed using the *fastx_trimmer* tool from the FASTX Toolkit (http://hannonlab.cshl.edu/fastx_toolkit/). After low-quality position trimming, the reads in which sequencing continued through the 3' adapter sequence (TGGAATTCTCGGGTGCCAAGG) were subjected to adapter clipping using the *fastx_clipper* tool from the FASTX Toolkit, and the reads shorter than 35 nt

were discarded. The reads were then subjected to two mapping rounds on the E14 mm9 genome reference. In the first round, the reads were mapped using Bowtie v1.0.0 [2], allowing for a maximum of 2 mismatches in the seed and allowing multiple mappings (parameters: *-n 2 -a -y --best*). In the second round, the unmapped reads were truncated to 35 nucleotides and mapped again with the same parameters used in the first round, except for the maximum allowed number of mismatches that was reduced to 1 (parameters: *-n 1 -a -y --best*). For transcriptome analysis, the full Ensembl mouse gene annotation was downloaded from UCSC (http://genome.ucsc.edu/cgi-bin/hgTables, Table: *ensGene*) in the BED format, and the transcript sequences were extracted from the reference E14 genome using the *fastaFromBed* utility from the BEDTools v2.17.0 suite [3] (http://code.google.com/p/bedtools/). Genome mappings corresponding to Ensembl annotated transcripts were extracted using custom Perl scripts, and converted to Ensembl transcriptome-based coordinates.

CIRS-seq analysis pipeline

First, SAM files with reads mapping for NT, DMS and CMCT conditions were sorted by transcript ID and position, then, using custom Perl scripts, the sum of reads mapping to each position was calculated. Since each CIRS-seq read gives information only on the base immediately preceding the first mapping position, which represents the reverse transcriptase stop point, we subtracted 1+ *n* (where *n* was the number of low-quality bases trimmed from reads 5'-end prior to mapping) to reads mapping positions to obtain the coordinates of the

RT stop point. Reads mapping to position 1 of transcripts were discarded from analysis since they represent the necessary stop point of reverse transcription. For DMS and CMCT reactivity scores calculation we adjusted the approach previously used by Kertesz and collegues for their PARS score [4]. Briefly, DMS and CMCT reactivity scores were defined as the $\log_2$ of the ratio between the normalized DMS (or CMCT) signal at a given position of the transcript, and the normalized signal in the NT sample at the same position. To normalize for the different sequencing depth between the DMS and the CMCT conditions with respect to the NT condition, we defined the normalization constants k_D and k_C as follows:

$$k_D = \frac{\left(\frac{(n_D + n_N)}{2}\right)}{n_D}$$

$$k_C = \frac{\left(\frac{(n_C + n_N)}{2}\right)}{n_C}$$

where n_N, n_D, and n_C are respectively the total number of mapped reads in the NT, DMS, and CMCT experiments. Then, normalized signals for DMS, and CMCT samples at position *i* of a given transcript were calculated as:

$$D_i = k_D \cdot n_{Di}$$

$$C_i = k_C \cdot n_{Ci}$$

where n_{Di} and n_{Ci} are respectively the raw reads count at position *i* in the DMS and CMCT samples.

Since we treated the DMS and CMCT conditions independently, two independent normalizing constants k_{N_D} and k_{N_C} were calculated to respectively normalize the NT condition with the DMS, and the CMCT treated samples as follows:

$$k_{N\_D} = \frac{\left(\frac{(n_N + n_D)}{2}\right)}{n_N}$$

$$k_{N\_C} = \frac{\left(\frac{(n_N + n_C)}{2}\right)}{n_N}$$

Similarly, the normalized signals for NT versus DMS, and NT versus CMCT samples at position *i* of a given transcript were calculated as:

$$N\_D_i = k_{N\_D} \cdot n_{Ni}$$

$$N\_C_i = k_{N\_C} \cdot n_{Ni}$$

where n_{Ni} is the raw reads count at position *i* in the NT sample.

DMS and CMCT scores at position *i* were then calculated as:

$$DMS_i = \max\left(\log_2\left(\frac{D_i + 1}{N\_D_i + 1}\right), 0\right)$$

$$\mathrm{CMCT}_i = \max\left(\log_2\left(\frac{\mathrm{C}_i + 1}{\mathrm{N\_C}_i + 1}\right), 0\right)$$

and all negative reactivity values were brought to zero.

Final score for position *i* was calculated as:

$$score_i = \max\left(DMS_i, CMCT_i\right)$$

Scores greater than zero, theoretically, represent transcripts positions reactive to either DMS or CMCT treatment, and so increasing scores are directly proportional to an higher probability of observing such positions in single-stranded conformation.

To obtain normalized reactivity, we performed a 90% Winsorising to remove outliers, by setting each score greater than the 90th percentile to the value of the 90th percentile, and then dividing each value by the 90th percentile to obtain a normalized reactivity ranging from 0 to 1. Positions with reactivities <0.3, 0.3-0.7, and >0.7 were considered respectively as weakly, moderately, and highly reactive.

Correlation between replicates

After calculating normalized reactivities for each transcript in the 2 biological replicates, Pearson correlation between replicates was calculated on the top

75th percentile of covered transcripts by averaging reactivities in 10nt window (sliding offset: 5nt) across each transcript. For individual transcripts analysis, the size of the window was reduced to 3nt (sliding offset: 1nt).

Protein-coding transcripts secondary structures analysis

For the analysis of protein-coding transcripts, we first defined a set of highly covered mRNAs by selecting all transcripts (including multiple isoforms of a single gene) in which at least 1 reverse transcriptase stop per nucleotide (RT-stops/nt) was observed in the DMS + CMCT treatments. Moreover, we performed the analysis on the last 50 nt of the 5'-UTR, the first 50 nt of the 3'-UTRs, and the first and last 100 nt of the coding region, and we discarded any transcript with 5'-/3'-UTR < 50 nt and coding region < 200 nt. This yielded a final list of approximately 9,500 transcripts. Per-base reactivity at position *i* was calculated as the average of the normalized reactivity for each transcript in the final mRNA dataset at position *i*. To calculate the complexive reactivities for the 5'-/3'-UTRs and coding region, we first averaged the reactivity values for each position across the analyzed region for all transcripts in our mRNA dataset to obtain a per-transcript regional mean value, and we then averaged the regional mean values across the entire dataset. The significance for the observed differences was calculated using a two-sided paired Wilcoxon rank sum test.

Comparison of protein coding transcripts and ncRNAs

Transcripts were classified according to the Ensembl annotation (http://genome.ucsc.edu/cgi-bin/hgTables, Table: *ensemblSource*). To enable inter-transcript comparison, we produced base-normalized reactivity scores. Since protein coding transcripts and ncRNAs differ in their GC% content, reactivities for A, C, G, and U residues were averaged independently. Mean reactivity scores for the 4 bases were then averaged to obtain the transcript's reactivity. To avoid biases due to different sequencing depths on different classes of transcripts, only the positions with coverage >50x were considered. Coverage per-base was calculated as the sum of the full length reads covering the base, and the RT-stops at the same position.

Lin28a binding sites analysis

For Lin28a binding sites analysis, the CLIP-Seq dataset GS37114 was downloaded from the Gene Expression Omnibus (GEO) database. Reads were clipped from adapter sequences, and mapped using Bowtie v1.0.0 to the same transcriptome reference used for CIRS-seq analysis. After peak-calling, summits were enlarged by 150 nt upstream and downstream, and average CIRS-seq reactivity was calculated. The significance for the observed differences was calculated using a two-sided Wilcoxon rank sum test.

Inference and representation of RNA secondary structures from reactivity scores

De novo secondary stuctures prediction was performed using RNAStructure v5.6 [5]. A SHAPE constraint file was generated by specifying the CIRS-seq reactivity at each position of the trascript. Positions at which no RT-stops occurred in either DMS or CMCT treatment were assigned a value of -500. Shape intercept and slope values were left as default (Intercept: -0.6 kcal/mol, Slope: 1.8 kcal/mol). RNAstructure CT files were pre-converted to dot-bracket notation using the *ct2dot* utility, and VARNA v3.9 [6] (http://varna.lri.fr/) was used to plot the graphical representations of the secondary structures starting from the structures in dot-bracket notation. For the prediction of Lin28a binding sites, we used the ViennaRNA Package v2.1.3 [7] (http://www.tbi.univie.ac.at/~ronny/RNA/index.html), and positions with reactivity >0.7 were constrained ("x"), while all other positions were left undefined ("."). We avoided unstable base-pairings by excluding lonely base-pairs and wobbled G:U base-pairs at the ends of helices (parameters: *--noLP --noClosingGU*).

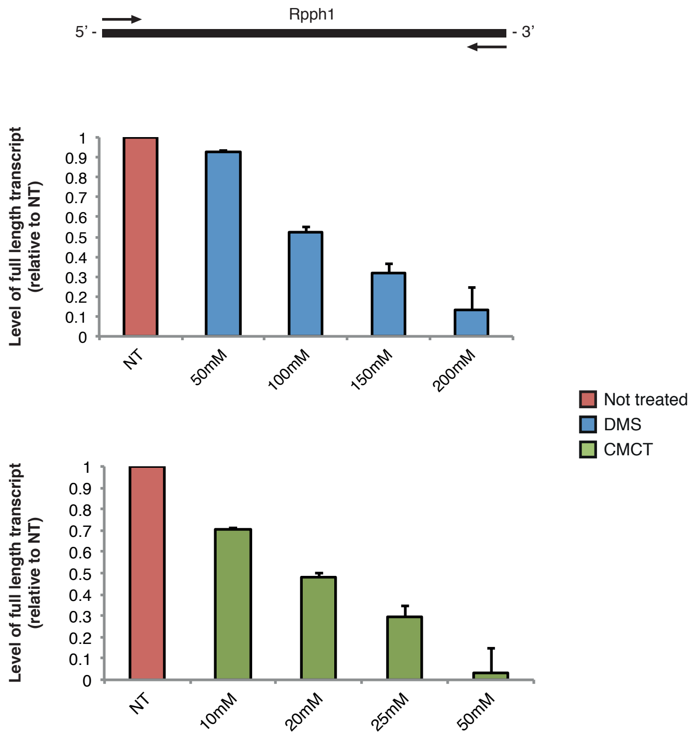

Rpph1
5' -
- 3'
Level of full length transcript (relative to NT)
1
0.9
0.8
0.7
0.6
0.5
0.4
0.3
0.2
0.1
0
NT
50mM
100mM
150mM
200mM
Not treated
DMS
CMCT
Level of full length transcript (relative to NT)
NT
10mM
20mM
25mM
50mM

Figure S1

Figure S1. Validation of DMS/CMCT modification efficiency at different chemical concentrations on a test transcript (Rpph1). The yield of the full-length transcript measured by RT-qPCR strongly decreases with increasing concentrations of DMS and CMCT. The concentrations were optimized to give a similar degree of modification with the two reagents.

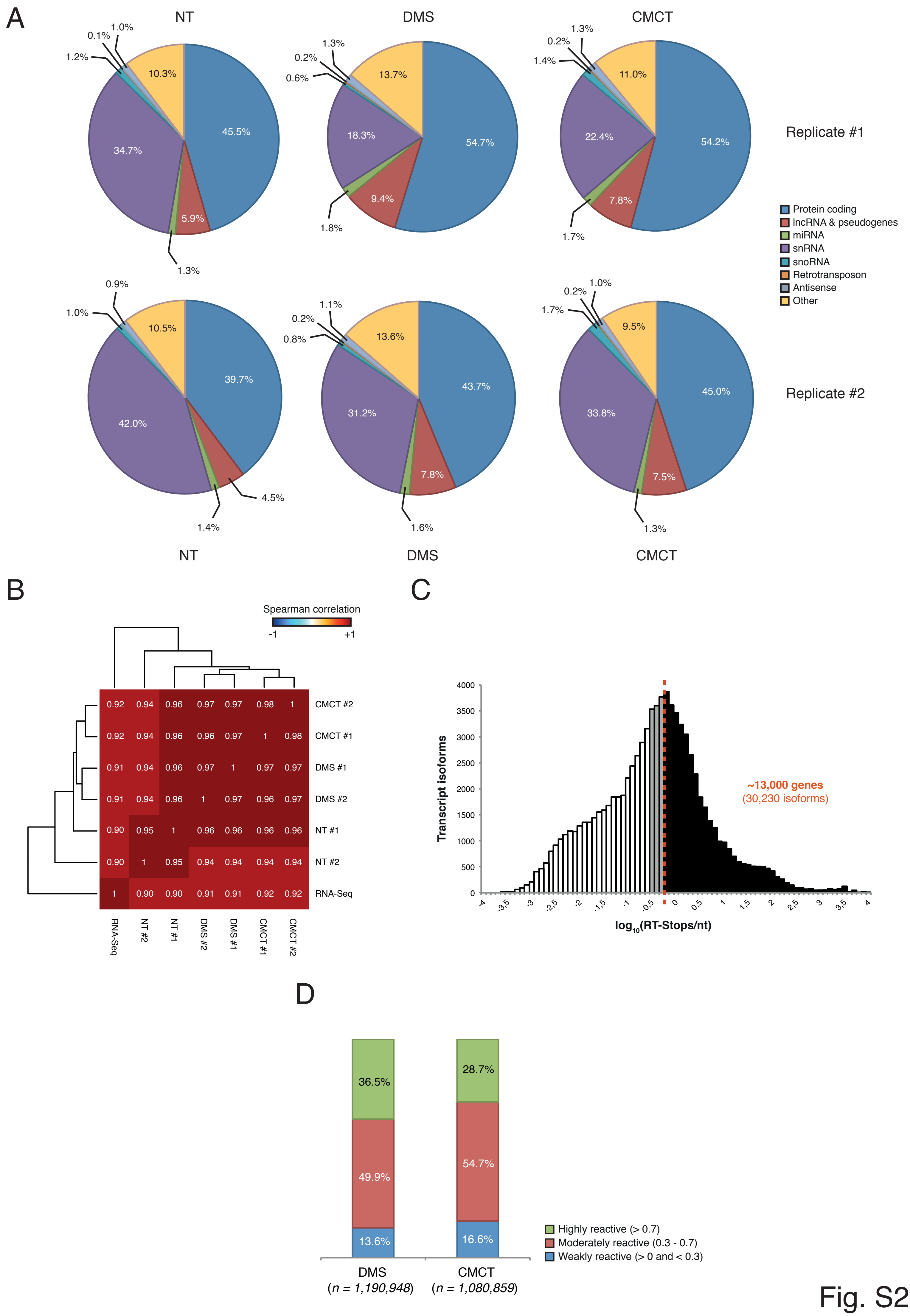
A
NT
DMS
CMCT
Replicate #1
Replicate #2
45.5%
34.7%
10.3%
5.9%
1.3%
1.2%
0.1%
1.0%
54.7%
18.3%
13.7%
9.4%
1.8%
0.6%
0.2%
1.3%
54.2%
22.4%
11.0%
7.8%
1.7%
1.4%
0.2%
1.3%
39.7%
42.0%
10.5%
4.5%
1.4%
1.0%
0.9%
43.7%
31.2%
13.6%
7.8%
1.6%
0.8%
0.2%
1.1%
45.0%
33.8%
9.5%
7.5%
1.3%
1.7%
0.2%
1.0%
Protein coding
lncRNA & pseudogenes
miRNA
snRNA
snoRNA
Retrotransposon
Antisense
Other
B
Spearman correlation
-1
+1
CMCT #2
CMCT #1
DMS #1
DMS #2
NT #1
NT #2
RNA-Seq
C
Transcript isoforms
log10(RT-Stops/nt)
~13,000 genes
(30,230 isoforms)
D
36.5%
49.9%
13.6%
28.7%
54.7%
16.6%
DMS
(n = 1,190,948)
CMCT
(n = 1,080,859)
Highly reactive (> 0.7)
Moderately reactive (0.3 - 0.7)
Weakly reactive (> 0 and < 0.3)

Fig. S2

Figure S2. CIRS-seq data statistics. (**A**) Distribution of reads mapping on each class of RNAs in the 3 samples of each biological replicate. (**B**) Heatmap of Spearman correlations between transcripts quantitation (RPKM) in the CIRS-seq and RNA-seq datasets. (**C**) Histogram showing the number of transcripts as a function of the average reverse transcriptase stops in the DMS/CMCT treatments divided by transcript length. Approximately 30,230 transcripts (~13,000 genes) have on average more than 1 RT-stop per nucleotide. (**D**) Percentages of bases recovered at different degrees of DMS/CMCT modification. Only the longer isoform for each gene was considered to avoid counting the same base multiple times.

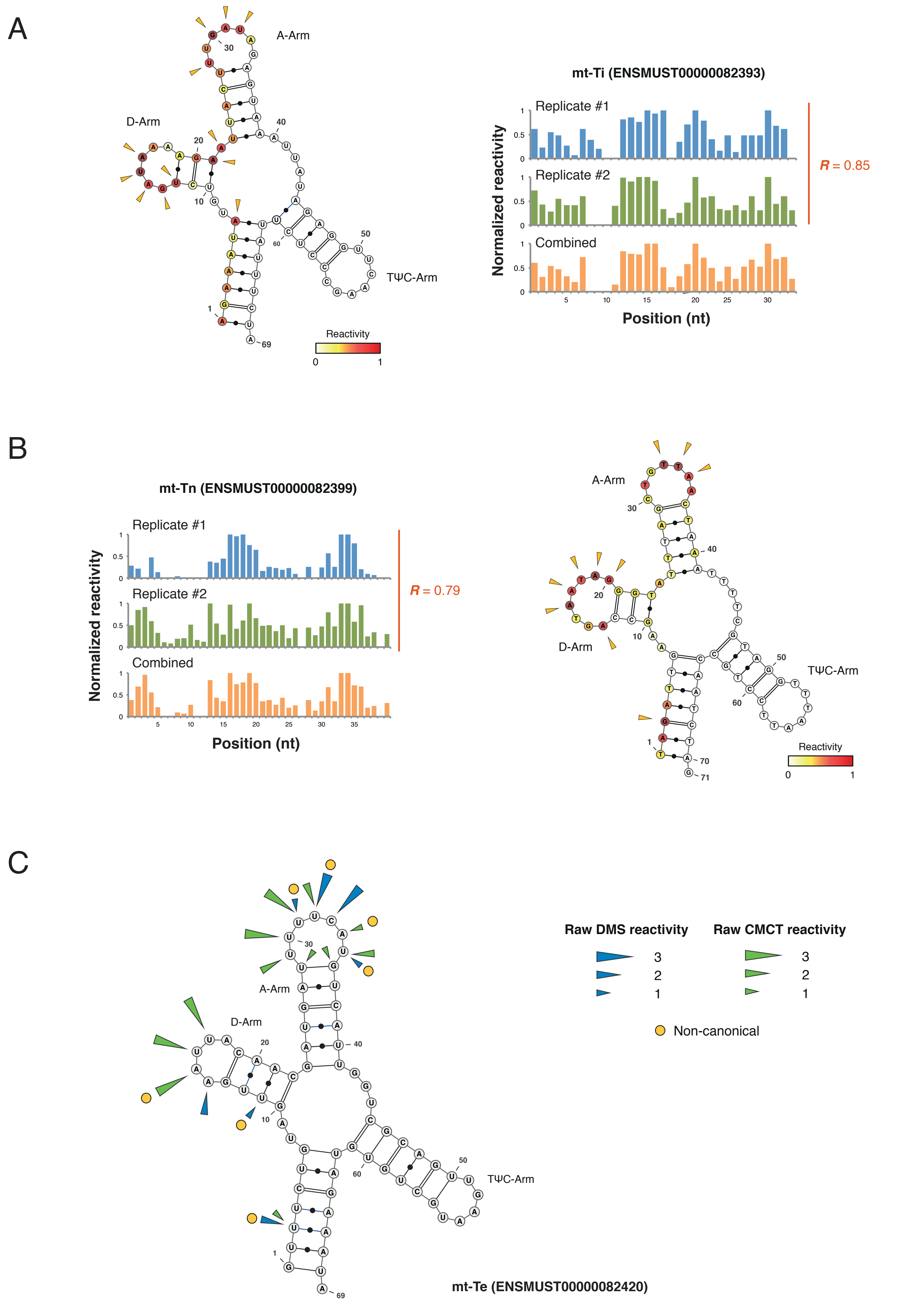
A
A-Arm
D-Arm
TΨC-Arm
Reactivity
0
1
mt-Ti (ENSMUST00000082393)
Replicate #1
Replicate #2
Combined
Normalized reactivity
Position (nt)
R = 0.85
B
mt-Tn (ENSMUST00000082399)
R = 0.79
C
Raw DMS reactivity
Raw CMCT reactivity
3
2
1
Non-canonical
mt-Te (ENSMUST00000082420)

Fig. S3

Figure S3. (**A**) Normalized reactivity profiles for the isoleucine tRNA and overlay of reactivity data on the phylogenetically derived secondary structure. Yellow arrows indicate highly reactive positions (reactivity > 0.7). Bases are color coded according to their reactivity. (**B**) Normalized reactivity profiles for the aspartic acid tRNA and overlay of reactivity data on the phylogenetically derived secondary structure. Yellow arrows indicate highly reactive positions (reactivity > 0.7). Bases are color coded according to their reactivity. (**C**) Raw DMS and CMCT reactivities for the glutamic acid tRNA (relative to Fig. 2C) and overlay of reactivity data on the phylogenetically derived secondary structure. Yellow circles indicate non-canonical reactivities for the two reagents.

A

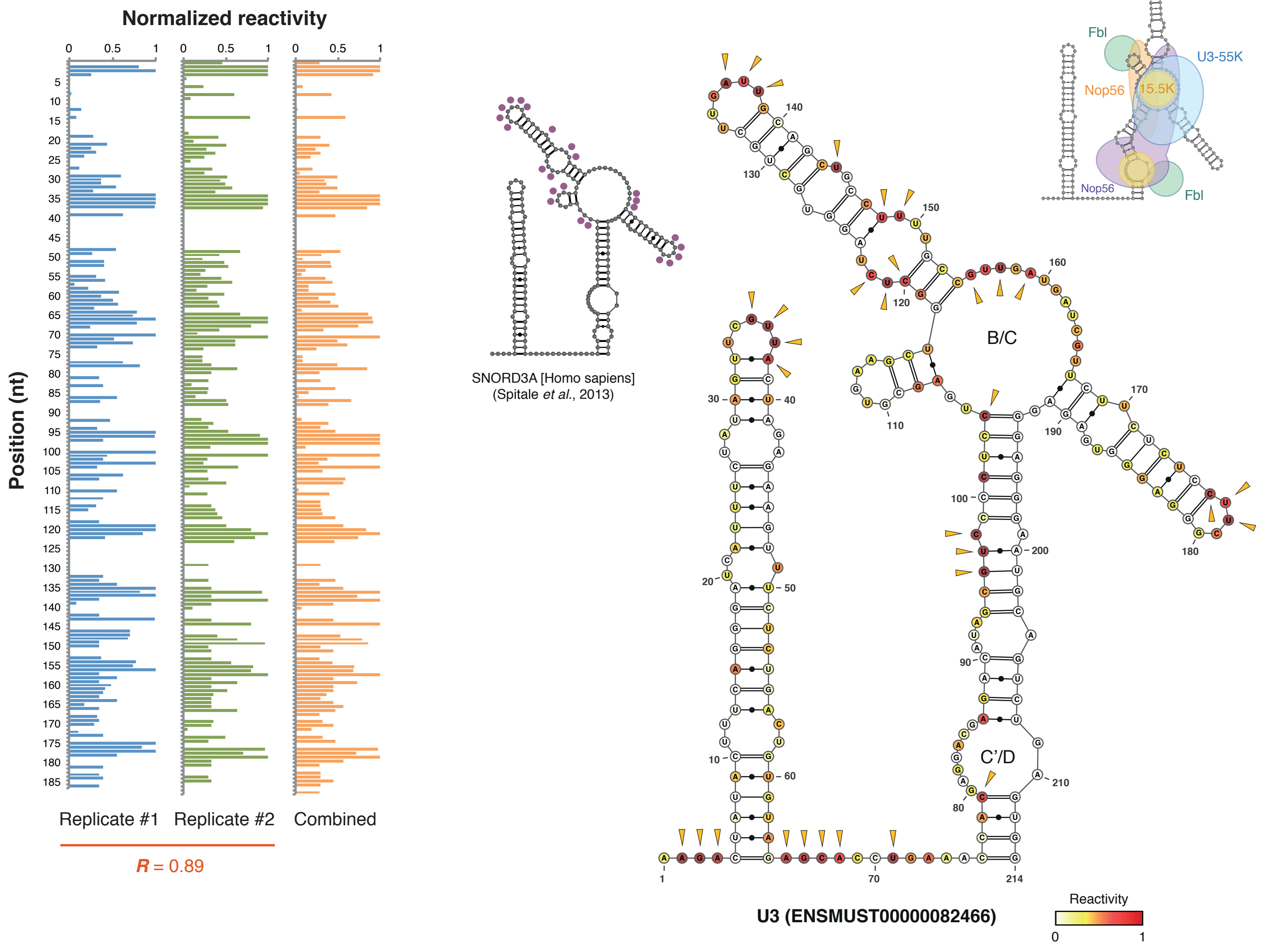
Normalized reactivity
Position (nt)
Replicate #1
Replicate #2
Combined
R = 0.89
SNORD3A [Homo sapiens]
(Spitale et al., 2013)
B/C
C'/D
U3 (ENSMUST00000082466)
Reactivity
Fbl
U3-55K
Nop56
15.5K

B

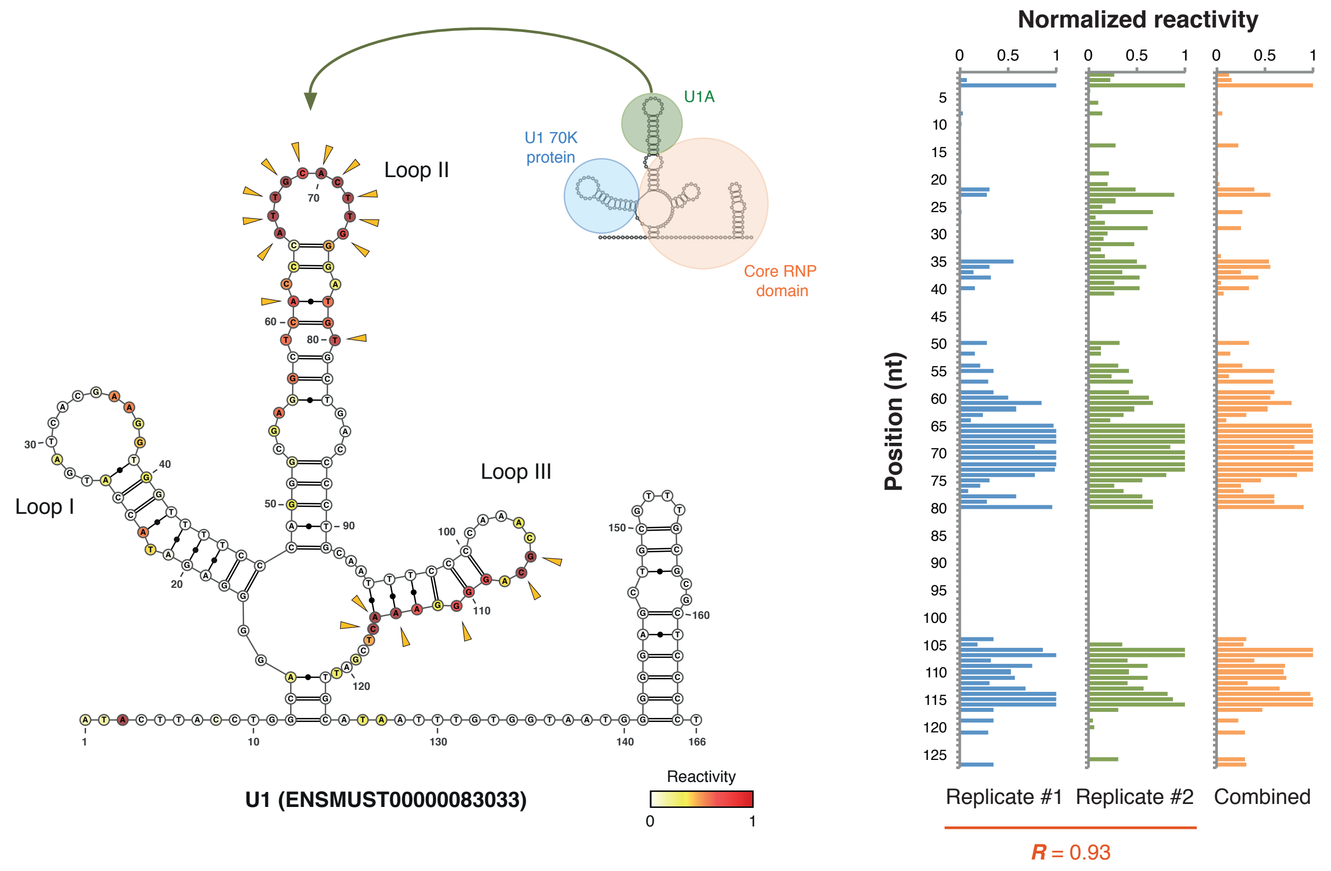
U1A
U1 70K
protein
Core RNP
domain
Loop II
Loop I
Loop III
U1 (ENSMUST00000083033)
Reactivity
Normalized reactivity
Position (nt)
Replicate #1
Replicate #2
Combined
R = 0.93

Fig. S4

Figure S4. (**A**) Normalized reactivity profiles for the U3 snoRNA and overlay of reactivity data on the phylogenetically derived secondary structure. Yellow arrows indicate highly reactive positions (reactivity > 0.7). Bases are color coded according to their reactivity. The structure of the human SNORD3A ortholog with superimposed SHAPE-reactive positions from [8] is also shown. (**B**) Normalized reactivity profiles for the U1 snRNA and overlay of reactivity data on the phylogenetically derived secondary structure. Yellow arrows indicate highly reactive positions (reactivity > 0.7). Bases are color coded according to their reactivity. Loop II of U1 snRNA is bound *in vivo* by the U1A protein, and the protein removal prior to chemical modification enables the high resolution of this domain.

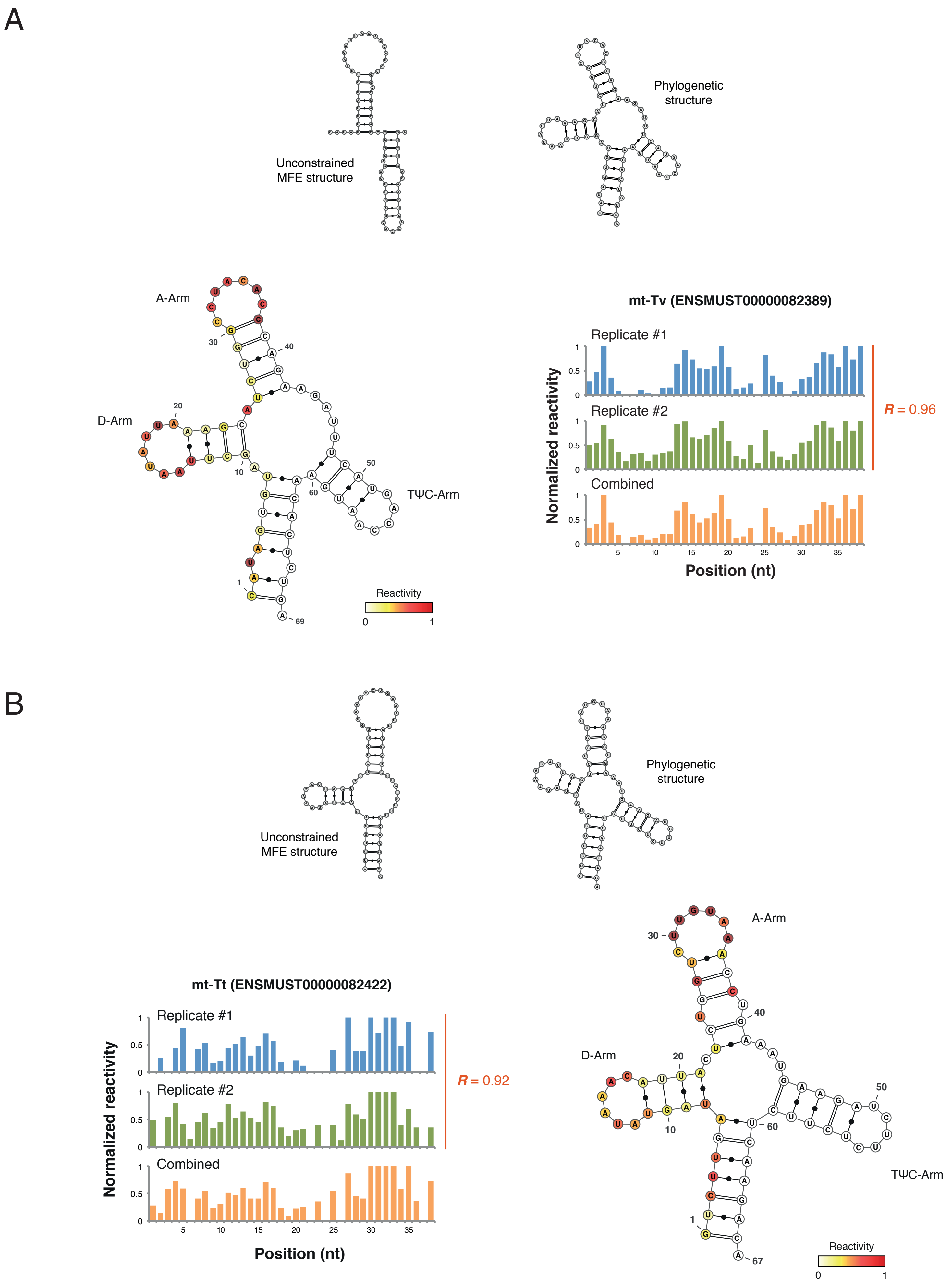
A
Unconstrained
MFE structure
Phylogenetic
structure
A-Arm
D-Arm
TΨC-Arm
Reactivity
0
1
mt-Tv (ENSMUST00000082389)
Replicate #1
Replicate #2
Combined
R = 0.96
Normalized reactivity
Position (nt)
B
Unconstrained
MFE structure
Phylogenetic
structure
mt-Tt (ENSMUST00000082422)
Replicate #1
Replicate #2
Combined
R = 0.92
Normalized reactivity
Position (nt)
A-Arm
D-Arm
TΨC-Arm
Reactivity
0
1

Fig. S5

Figure S5. (**A**) Normalized reactivity profiles for the valine tRNA and overlay of reactivity data on the secondary structure inferred from chemical constraints. Bases are color coded according to their reactivity. The structure of the phylogenetically derived and unconstrained MFE structures are also shown. (**B**) Normalized reactivity profiles for the threonine tRNA and overlay of reactivity data on the secondary structure inferred from chemical constraints. Bases are color coded according to their reactivity. The structure of the phylogenetically derived and unconstrained MFE structures are also shown.

Primer	Sequence (5' -> 3')
RT-Rpph1-For	AGTGGGCGGAGGAAGCTCAT
RT-Rpph1-Rev	AATGGGCGGAGGAGAGTAGTCTGA
CIRS RC5 Adapter (3' Adapter)	(P)-GATCGTCGGACTGTAGAACTCTGAAC-(C3)
CIRS RC3 Adapter (5' Adapter)	CCTTGGCACCCGAGAATTCCA
Illumina PCR For	AATGATACGGCGACCACCGAGATCTACACGTT CAGAGTTCTACAGTCCGA
Illumina Indexed PCR Rev	CAAGCAGAAGACGGCATACGAGATNNNNNNGT GACTGGAGTTCCTTGGCACCCGAGAATTCCA

Table S1. Sequence of primers used in this study.

References

1. Incarnato D, Krepelova A, Neri F: **High-throughput single nucleotide variant discovery in E14 mouse embryonic stem cells provides a new reference genome assembly.** *Genomics* 2014.

2. Langmead B, Trapnell C, Pop M, Salzberg SL: **Ultrafast and memory-efficient alignment of short DNA sequences to the human genome.** *Genome Biol* 2009, **10**:R25.

3. Quinlan AR, Hall IM: **BEDTools: a flexible suite of utilities for comparing genomic features.** *Bioinformatics* 2010, **26**:841–842.

4. Kertesz M, Wan Y, Mazor E, Rinn JL, Nutter RC, Chang HY, Segal E: **Genome-wide measurement of RNA secondary structure in yeast.** *Nature* 2010, **467**:103–107.

5. Reuter JS, Mathews DH: **RNAstructure: software for RNA secondary structure prediction and analysis.** *BMC Bioinformatics* 2010, **11**:129.

6. Darty K, Denise A, Ponty Y: **VARNA: Interactive drawing and editing of the RNA secondary structure.** *Bioinformatics* 2009, **25**:1974–1975.

7. Lorenz R, Bernhart SH, Höner Zu Siederdissen C, Tafer H, Flamm C, Stadler PF, Hofacker IL: **ViennaRNA Package 2.0.** *Algorithms Mol Biol* 2011, **6**:26.

8. Spitale RC, Crisalli P, Flynn RA, Torre EA, Kool ET, Chang HY: **RNA SHAPE analysis in living cells.** *Nature Chemical Biology* 2013, **9**:18–20.
